# Differential Anxiety Responses in Acute Myocardial Infarction vs. COVID-19 Pneumonia Patients

**DOI:** 10.3390/medicina60060902

**Published:** 2024-05-29

**Authors:** Sezgin Kehaya, Bilkay Serez Kaya, İlker Yilmam, Muhammet Gürdoğan

**Affiliations:** 1 Department of Neurology, Faculty of Medicine, Trakya University, Edirne 22030, Turkey; 2 Department of Chest Medicine, Faculty of Medicine, Trakya University, Edirne 22030, Turkey; bilkayserez@trakya.edu.tr (B.S.K.); drilkeryilmam@gmail.com (İ.Y.); 3 Department of Cardiology, Faculty of Medicine, Trakya University, Edirne 22030, Turkey; drmgurdogan@gmail.com

**Keywords:** COVID-19 pneumonia, myocardial infarction, death anxiety, Beck anxiety inventory, coronavirus anxiety, Turkey

## Abstract

*Background and Objectives*: Amidst the COVID-19 pandemic, concerns about the psychological impact of disease-related anxiety on public health have risen. This study aims to compare general and death anxiety levels between acute coronary artery syndrome and COVID-19 pneumonia patients. *Materials and Methods*: A cross-sectional study of 132 individuals, including acute myocardial infarction (MI), COVID-19 pneumonia patients, and healthy volunteers from Trakya University Hospital (Turkey), was analyzed. Validated scales like the Beck Anxiety Inventory (BAI), Coronavirus Anxiety Scale Short Form, and Thorson–Powell Death Anxiety Scale (TPDAS) were employed. Demographic data such as age, gender, income levels, employment status, presence of a close relative with COVID-19, and whether participants followed COVID-19-related news were collected and compared across groups with significance level of 0.05 set for all analyses. *Results*: Among 41 COVID-19, 41 MI, and 50 healthy subjects, the pneumonia group showed highest COVID-19 anxiety (*p* = 0.01) and BAI scores (*p* = 0.008). Both COVID-19 and MI patients had significantly higher BAI and TPDAS scores compared to healthy controls (*p* < 0.001). Factors like female gender (*p* = 0.004), low education (*p* = 0.003), current employment (*p* = 0.008), and low income (*p* = 0.002) correlated with higher BAI scores. Low income (*p* = 0.001) and COVID-19 news exposure (*p* = 0.002) correlated with higher TPDAS scores. Males and married patients had lower anxiety scores (*p* = 0.008). High income, education, and employment reduced anxiety levels (*p* = 0.008). TPDAS scores decreased with higher income (*p* = 0.001), but increased in the MI group (*p* = 0.002) with COVID-19 news exposure. The multivariate linear regression analysis found that MI and COVID-19 pneumonia were associated with TPDAS; female gender, university education, and COVID-19 pneumonia with the Beck scale; and COVID-19 pneumonia with anxiety scores on the COVID-19 Anxiety scale. *Conclusions*: This research showcases differing anxiety patterns between illnesses such as MI and COVID-19 pneumonia amidst the pandemic, emphasizing the amplifying influence of media coverage on death-related anxieties. It underscores the imperative of targeted interventions and socioeconomic considerations in managing psychological consequences and formulating responsive public health strategies.

## 1. Introduction

During the COVID-19 pandemic caused by SARS-CoV-2, almost all countries reported constant death rates in the news. Many countries implemented strict precautions to curb the spread of the disease. Closures disrupted the general flow of life. Activities such as education, work, and essential grocery shopping shifted online, altering the way of life. The concept of a “new normal” emerged.

The pandemic’s economic losses caused global concerns regarding plans such as careers, vacations, and expectations. The fear of contracting a deadly disease and worries about the future can create internal conflicts in individuals. People’s behaviors may change in response to this anxiety, leading to negative attitudes such as looting, social isolation, and reluctance to help, which we observed in various parts of the world [1,2]. The opening of units isolating patients, like pandemic services, and their coverage in the news further amplifies disease anxiety and, consequently, in turn, increases general anxiety and fear of death among individuals. Avoidance of seeking medical care by patients and disruption of the follow-up of chronic diseases also affect public health.

If the entire society falls ill simultaneously, healthcare services worldwide will be insufficient. Therefore, taking precautions is logical. However, broadcasting death news on all television channels and inducing fear of death across society can have negative effects on public health. It is known that fear of death can lead to irrational behaviors, including suicide [2]. Heart attack is the disease with the highest fear of death. According to WHO data, ischemic heart disease has the highest mortality rate [3]. Although COVID-19 pneumonia creates fear of death, its mortality rates are lower. A study before the COVID-19 pandemic comparing death anxiety between myocardial infarction (MI) and cancer reported death anxiety to be higher in the former [4]. However, during the pandemic, the level of anxiety may vary among patients.

During the pandemic period, we aimed to compare general anxiety and anxiety of death in patients with acute coronary artery disease and the COVID-19 pneumonia. This study investigates the impact of risk factors such as age, gender, news consumption, income, and education level on death anxiety in MI and COVID-19 pneumonia, as well as overall anxiety levels. Understanding these factors is crucial for developing targeted interventions to reduce anxiety and improve mental health outcomes in patients with serious health conditions. The findings can guide healthcare professionals in providing more personalized care and support to those at higher risk of anxiety. Identifying fear of death and its influencing factors in times of heightened societal anxiety can guide interventions in subsequent events.

## 2. Materials and Methods

After obtaining governmental permission from the Health Ministry on studies for COVID-19 infection (T.C. Sağlık Bakanlığı Sağlık Hizmetleri Genel Müdürlüğü form: Bilkay Serez-2021-01-01T16_46_59) and approval by Trakya University Non-Interventional Studies Ethical Committee (approval date: 18 January 2021. TÜTF-BAEK 2021/27 no: 02/09), we included 132 subjects in our study between 1 February 2021 and 1 August 2022. All subjects gave their informed consent for inclusion before they participated in the study. The study was conducted in accordance with the Declaration of Helsinki. Taking the study conducted by Şahan et al. as a reference for the power analysis, the effect size was calculated as 0.29 [4]. Considering the calculated effect size, it was decided to include a total of 120 volunteer participants, 40 in each group, in the study, which was planned as 3 independent groups with a significance level of 5% and power of 80%. Our population comprised 41 acute myocardial infarction (MI) patients, 41 COVID-19 pneumonia patients, and 50 healthy volunteers. All participants were collected from Trakya University Hospital. After ensuring a safe environment, patients were evaluated for delirium by a neurologist, and information about the study was provided. Informed consent was obtained from those who chose to participate, and their signatures were collected. Participants were selected from healthy caregivers accompanying individuals to neurology or cardiology outpatient clinics, the COVID-19 pneumonia pandemic service, and the coronary intensive care unit. Those under 18 years of age, individuals without education, those with a history of psychiatric illness, those using psychotropic drugs (including SSRIs), and those with signs suggesting brain damage related to the central nervous system such as stroke, trauma, and mass were not included in the study.

The study population was divided into three groups. The first group comprised healthy volunteers who tested negative for COVID-19 PCR or had no history of COVID-19 infection. The participants were not obligated to test for COVID-19 PCR but were questioned for any symptoms. Suspicion for the disease or previous positive PCR testing was used for exclusion criteria to the study. The second group included patients receiving treatment for COVID-19 pneumonia, who tested positive for COVID-19 PCR and exhibited radiological findings consistent with COVID-19 pneumonia on chest X-rays or computed tomography scans. The study did not include individuals who tested negative for PCR or did not have radiological findings consistent with COVID-19 pneumonia. The third group consisted of patients presenting with acute coronary syndrome and elevated troponin levels within the first seven days, with or without ST elevation, indicative of acute myocardial infarction. The study did not include patients whose symptoms persisted beyond seven days or were diagnosed as angina pectoris. Also, those who tested positive for COVID-19 PCR, which was obligated before attending to coronary unit, were excluded from the study.

Similar to previous studies, the demographic characteristics of participants were determined [2,4]. These included age, gender (female, male, or other), educational status (divided into three groups: 8 years, 12 years, university), income level (categorized into low income for current minimum wage or lower values, medium income for values up to twice the minimum wage, and high income for values exceeding twice the minimum wage), marital status (married or unmarried, including divorced, widowed, or never married individuals), employment status (categorized into three groups: actively employed, unemployed, and retired), presence of a close relative who had contracted COVID-19, and whether the participant followed COVID-19 news.

Participants’ general anxiety levels were assessed using the Turkish Version of the Beck Anxiety Inventory, which has previously undergone validity and reliability studies in Turkey [5]. This self-report questionnaire consists of 21 items, where individuals rate their symptoms over the past week on a scale of 0 = not at all, 1 = mild, 2 = moderate, to 3 = severe, yielding scores between 0 and 63. Anxiety severity is categorized based on scores: 8–15, indicating mild anxiety symptoms; 16–25, indicating moderate anxiety; and 26–63, indicating severe anxiety. Higher scores correspond to higher levels of anxiety. Participants’ anxiety presence and severity were compared based on the scores obtained from the scale.

For assessing participants’ COVID-19 anxiety levels, the Coronavirus Anxiety Scale Short Form was utilized, which has previously undergone validity and reliability studies in Turkey [6]. This 5-item test asks individuals to rate their personal experiences over the past two weeks on a scale of 0 = never, 1 = rarely, 2 = some days, 3 = more than seven days, to 4 = nearly every day, yielding scores between 0 and 20. Higher scores indicate higher anxiety levels, and numerical comparisons were made in our study based on these scores.

To evaluate participants’ death anxiety levels, the Thorson–Powell Death Anxiety Scale Revised was employed, which has previously undergone validity and reliability studies in Turkey [7]. This 25-item scale includes positive and negative statements, to which participants respond with degrees of agreement/disagreement ranging from very suitable, suitable, undecided, unsuitable, to very unsuitable. Positive questions are scored from 0 to 4, while negative questions are scored from 4 to 0, resulting in scores ranging from 0 to 100. Higher scores indicate higher levels of death anxiety, and numerical comparisons were made in our study based on these scores.

Statistical analysis was conducted using IBM SPSS V22.0. The assumption of normal distribution was assessed using the Shapiro–Wilk test. For variables that met the assumption of normality, one-way analysis of variance (ANOVA) was employed for comparisons between more than two independent groups. The Kruskal–Wallis test was used for variables that did not follow a normal distribution. In the event of significant differences found in comparisons between more than two independent groups, appropriate post hoc tests (such as Tukey or Dunn–Bonferroni) were performed. Factors with a *p*-value < 0.2 in the univariate regression analysis were included in the multivariate linear regression analysis. Relationships between qualitative variables were investigated using chi-square tests. Descriptive statistics, including mean and standard deviation or median and quartiles, are provided for quantitative variables, while frequency and percentage are presented for qualitative variables. A significance level of 0.05 was set for all statistical analyses.

## 3. Results

From 1 February 2021 to 1 August 2022, we proposed that patients be included in our study. Nearly three of four COVID-19 pneumonia and MI patients refused to speak about death. Half of the healthy volunteers refused. Our population included 41 COVID-19, 41 MI, and 50 healthy subjects. Table 1 shows the demographic data of the patients. Because of low attendance to our study, we could not match age, income, and working status between the groups. Instead, we performed regression analysis. MI patients were older, and COVID-19 pneumonia patients were more prevalent in the low-income group. COVID-19 anxiety and general anxiety levels were highest in the pneumonia group. There was no difference between COVID-19 and MI patients according to Beck anxiety and Thorson–Powell death anxiety scores, but they had significantly higher scores than the healthy group.

Table 2 shows the distribution and associations of anxiety levels among the predefined risk factors. Thorson–Powell scores were normally distributed during statistical analysis, but because it is an ordinal Likert scale, we used nonparametric tests. COVID-19 anxiety and Beck depression scores were not distributed normally, and Mann–Whitney U and Kruskal–Wallis tests were used. Females had statistically higher Beck anxiety scores. A low level of education was associated with statistically significant higher scores for general anxiety and nonstatistically higher death anxiety scores. High income was inversely related to death and general anxiety. Current workers had lower death and general anxiety levels. COVID-19 news followers had statistically higher death anxiety scores but equal general anxiety scores. Death anxiety and general anxiety scores were alike between MI and COVID-19 pneumonia patients and lower in healthy subjects.

Factors with a *p*-value < 0.2 were included in the multivariate linear regression analysis (Table 3). The multivariate linear regression analysis showed that MI and COVID-19 pneumonia were associated with higher TPDAS scores; female gender and COVID-19 pneumonia with higher, but university education with lower, scores in the Beck scale; and only COVID-19 pneumonia was associated with anxiety scores on the COVID-19 Anxiety scale statistically.

**Figure 1:**
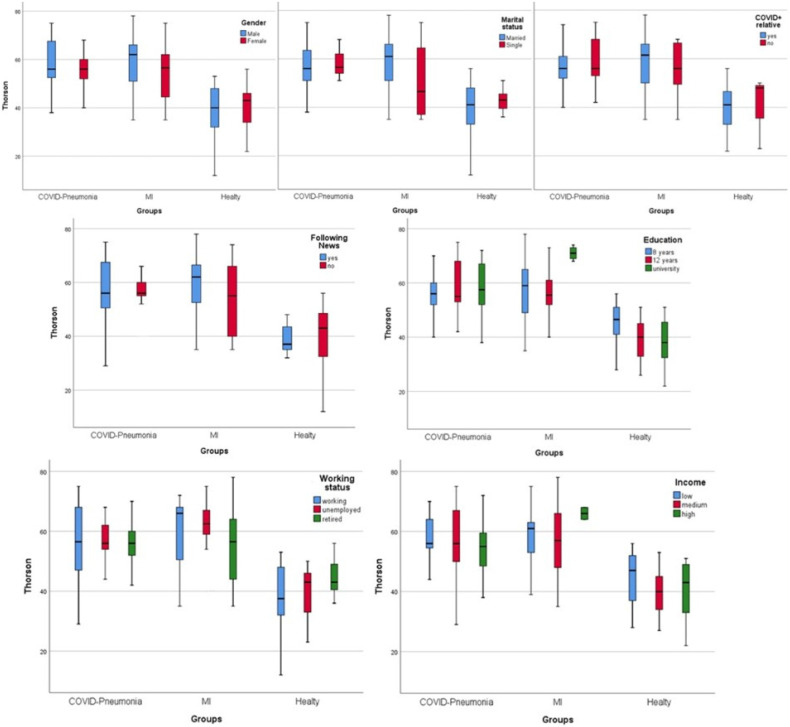
Thorson–Powell scores between groups.

Death anxiety levels measured by Thorson–Powell inquiry in the three groups according to other risk factors are shown in Figure 1. Death anxiety scores decreased linearly by increased income in the pneumonia and healthy groups but increased in the MI group (*p* = 0.001). COVID-19 news followers had higher scores only in the MI group (*p* = 0.002). We did not observe statistical differences between groups according to gender, marital status, and known COVID-19 infection relative. However, married males had higher death anxiety scores in the MI group. Death anxiety scores increased in the MI group with education level (*p* < 0.001). Workers had lower scores than retired and healthy groups.

**Figure 2:**
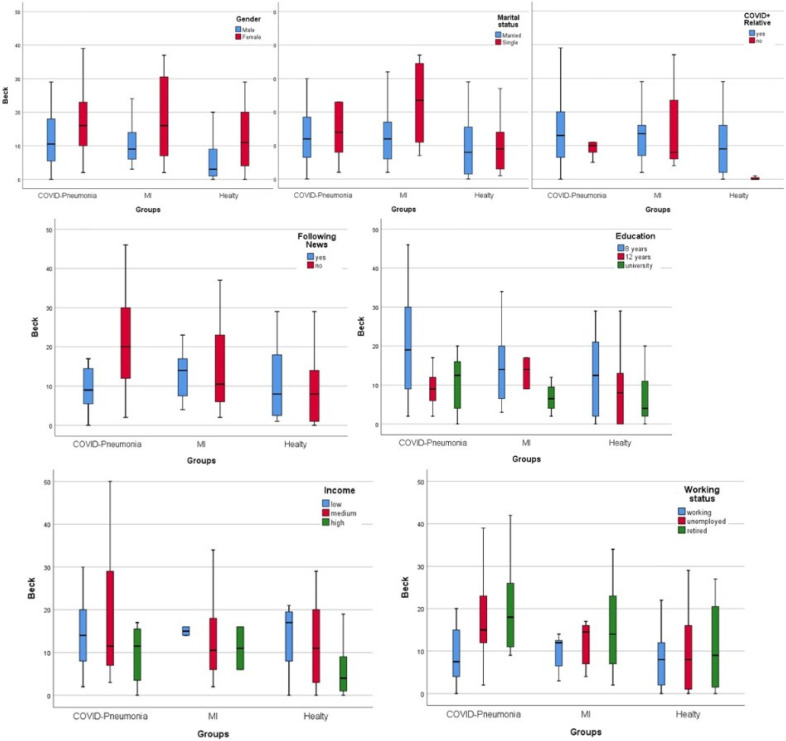
Beck anxiety scores between groups.

General anxiety, measured by the Beck Depression Inventory, was compared with risk factors between groups and is shown in Figure 2. Males and married patients showed lower anxiety scores than females and singles in all groups (*p* = 0.008). Patients following COVID-19 news had significantly less anxiety than nonfollowers only in the COVID-19 pneumonia group (*p* = 0.003). Having a COVID-positive relative increased scores nonsignificantly in COVID-19 pneumonia and healthy groups but not in the MI group. High income and having a current job decrease anxiety levels in all groups (*p* = 0.008). With increasing levels of education, anxiety levels decreased.

Because of very low scores in the COVID-19 Anxiety Inventory, no statistical data were obtained between the groups.

In summary, COVID-19 anxiety was found to be elevated only in the COVID-19 pneumonia group. Beck Anxiety Inventory and Thorson–Powell death anxiety scores were higher in both MI and COVID-19 pneumonia groups compared to healthy controls. While females exhibited higher Beck anxiety scores, similar trends were observed in Thorson–Powell death anxiety scores. Low educational attainment was statistically associated with higher Beck anxiety scores, although this association was not observed for Thorson–Powell scores; however, the highest Thorson–Powell scores were still observed in individuals with lower education levels. An increase in income was statistically associated with decreased Beck and Thorson–Powell scores. Following COVID-19 news did not alter Beck scores but was associated with increasing Thorson–Powell scores. An increase in income was associated with an increase in Thorson–Powell scores in both healthy and COVID-19 groups, while this relationship was not observed in the MI group. Higher Thorson–Powell scores were observed in males and married individuals across all groups. In the MI group, following COVID-19 news was associated with increased Thorson–Powell scores. An increase in education was associated with an increase in Thorson–Powell scores only in the MI group. Male gender, marital status, employment status, and higher educational attainment were associated with lower Beck scores across all groups. Following news was only associated with lower Beck scores in the COVID-19 group.

## 4. Discussion

In our study, COVID-19 anxiety was only observed in the COVID-19 pneumonia group. High Beck anxiety scores were associated with female gender and lower educational levels. In contrast, low anxiety scores were associated with male gender, higher income, higher educational attainment, marital status, and active employment. High Thorson–Powell scores were associated with lower educational levels, lower income, and following COVID-19 news, while low Thorson–Powell scores were associated with higher income and employment. An increase in income was related to low Thorson–Powell scores in both healthy and COVID-19 pneumonia groups but not in the MI group. Education increased Thorson–Powell scores in the MI group but not in the others. Watching news increased Thorson–Powell scores in the MI group and decreased Beck scores in the COVID-19 pneumonia group.

The World Health Organization has reported deteriorations in mental health during the pandemic, including increases in anxiety, depression, and even suicides [8]. The Coronavirus Anxiety Scale has been validated and used in many countries worldwide [9]. Although we were unable to conduct statistical analysis due to very low scores, our findings of higher scores in the COVID-19 pneumonia group compared to both the myocardial infarction group and healthy individuals lead us to speculate that these patients may be experiencing coronaphobia due to disease-specific anxiety, which was previously attempted to explain for depression, generalized anxiety, and death anxiety [2]. The reason might be the utility of this scale in different populations in clinical settings. A systematic review and meta-analysis reported the scale’s validity but speculated about different methodologies of previous studies [9].

The pandemic period has witnessed a significant increase in anxiety levels across all societies worldwide [10]. This phenomenon has been consistently associated with factors such as female gender, low educational level, low income, living alone, and unemployment, as confirmed by numerous studies [10,11]. The gender disparity in anxiety, which existed even before the pandemic, has become even more pronounced during this period. Factors such as women’s socioeconomic status, their engagement in lower-paying jobs, and their increased responsibilities in household management may have exacerbated their home situations during lockdown periods. Anxiety, recognized as a global health issue, is estimated to have caused 44.5 million (30.2 to 62.5) disability-adjusted life years (DALYs) worldwide in 2020 [10]. Higher levels of anxiety have been reported among hospitalized patients, with those admitted due to COVID-19 exhibiting higher anxiety scores compared to those hospitalized for myocardial infarction, pneumonia, and intensive care stays [12]. In this study, anxiety levels were found to be higher in all patients hospitalized due to COVID-19 compared to healthy volunteers, and they were also higher in patients hospitalized for COVID-19 compared to those admitted for other diseases. A study conducted in China reported longer hospital stays and worse outcomes for patients with anxiety and depression, indicating a more severe course of COVID-19 in these individuals [13]. Previous studies have also noted worse outcomes for patients with anxiety who experience myocardial infarction [14]. The presence of anxiety is associated with postpartum depression during the COVID-19 period [15]. These findings underscore the importance of addressing anxiety as a public health issue previously, particularly considering its potentially greater impact on specific populations [11,12].

Death anxiety scales have been studied in different cultures, yielding similar results. Factors such as advancing age, exposure to death, sociocultural background, and religious affiliation have been associated with death anxiety. Similarly, in our study, we found associations between gender, education, and income levels with death anxiety. While we did not directly investigate beliefs in our study, all participants indicated their belief in life after death when asked about the Death Anxiety Scale. A study examined death anxiety among emergency workers who frequently confront death, finding higher levels among females and lower levels among those with higher education and experience [16]. Another study conducted an online survey on death anxiety, finding no significant effect of religiosity but identifying cognitive–emotional, gender, and sociocultural factors as predictors [17]. Jong J. conducted a meta-analysis comparing religiosity and death anxiety, suggesting a U-shaped relationship wherein religious and nonreligious individuals experience lower levels of anxiety. In comparison, uncertainty about death is associated with higher anxiety levels [18]. Additionally, their data suggested that death anxiety can lead to increased religiosity according to terror management theory [18,19].

It has been demonstrated that there are variations in anxiety levels among patient groups. For example, in a comparison between two potentially fatal diseases, such as cancer and MI, higher levels of death anxiety have been reported in MI patients [4]. It is natural to expect an increase in death anxiety during processes like the pandemic, which serve as reminders of mortality. Throughout the pandemic, numerous studies have been conducted using various scales to investigate death anxiety. Yang et al. investigated death anxiety during COVID-19 and its related factors among elderly Chinese people, reporting marital status as a predictive factor for death anxiety [20]. They supported both the vulnerability–stress model and terror management theory regarding death anxiety. In the UK, resilience during the beginning of the COVID-19 pandemic was studied, and death anxiety was associated with nonresilience, similar to findings in [21]. In another study during the pandemic, resilience was associated with death anxiety, while death anxiety was also linked to gender and employment status [22]. In Turkey, a study conducted during the COVID-19 pandemic found associations between death anxiety, gender, and education level in the general population [23]. In Pakistan, a study reported that death anxiety played a mediating role in the relationship between stigmatization and panic disorder during the COVID-19 pandemic, with age and gender as contributing factors [24]. Waite et al. reported death anxiety during the pandemic as a mental health issue related to other psychopathologies such as paranoia, depression, and anxiety [25]. They compared anxiety levels and other determinants between the first and second waves of the pandemic, which were one month apart, and observed relationships between these factors. They identified gender and low income as risk factors for high death anxiety, consistent with our findings. Özgüç et al., in a meta-analysis, demonstrated the relationship between death anxiety and sociodemographic factors such as fear of COVID-19, gender, and occupation, which aligns with the findings of our study [26].

Ahmet Çağlar et al. compared COVID-19 pneumonia and acute myocardial infarction patients who presented to the emergency department during the pandemic with a healthy control group [27]. They reported similar levels of death anxiety between the COVID-19 pneumonia and MI groups. However, they noted that a neurological examination was not performed, and patients with a Glasgow Coma Score of 15 were included in the study. A study reported that exposure to information, fear of contagion, and symptoms of hypochondriasis were associated with death anxiety during the COVID-19 pandemic [28]. Another study investigated the relationship between death anxiety and media involvement, empathy, sympathy, and affect and found that media involvement was positively associated with death anxiety [29]. In our study, we also found higher levels of death anxiety associated with COVID-19 news exposure in our study population. However, this increase was observed only in the MI group, while it was similar regarding news exposure between the pneumonia and healthy groups. Beck anxiety scores increased with news exposure in the MI group, while they decreased in the COVID-19 pneumonia group. Moreover, death anxiety increased with higher levels of education in the MI group. The decrease in anxiety in COVID-19 pneumonia and the increase in death anxiety with higher education levels in the MI group suggest the fact that MI has higher fatality rates than COVID-19 pneumonia, and this information could be obtained through education.

One of the notable strengths of our study lies in its focus on a particularly challenging and specialized patient group, namely, those suffering from COVID-19 pneumonia and requiring intensive care treatment in isolation from the wider community and other patients. This group is known to have high mortality rates, making it a crucial area of investigation during the pandemic. The same is true for myocardial infarction patients at the coronary intensive unit, which has high mortality rates.

However, it is important to acknowledge certain limitations of our study. These include the relatively small number of patients involved and the fact that the study was conducted at a single center within one country. Because of low attendance to our study, we could not match age, income, and working status between the groups. Additionally, because participation was voluntary and only patients who gave consent were included, there is a possibility of selection bias, meaning that the findings may not fully represent the entire population of patients with COVID-19 pneumonia and MI.

Despite its limitations, our study aligns with previous findings, suggesting that an individual’s existing anxiety level can increase further when exposed to alarming information, even if it is not accurate. We observed that death anxiety intensifies in the presence of illnesses, which in turn can elevate overall anxiety levels. Additionally, our results indicate that higher education levels are associated with a reduction in unfounded anxiety. We believe that these insights are generalizable to the broader population, highlighting the importance of addressing anxiety through accurate information and education.

Based on our data, it is crucial for media and society to carefully share information that could create unfounded anxiety, particularly among individuals with illnesses, low income, or low education levels. If necessary, governments should appropriately restrict the dissemination of misleading information and ensure that accurate, educational, and rational information is accessible. This approach could play a significant role in protecting the mental and emotional health of the public during future pandemics. Additionally, we believe that healthcare workers adopting an educational approach with patients can help reduce both death-related and general anxiety.

## 5. Conclusions

In conclusion, in our study conducted during the pandemic period, we assessed the levels of general anxiety and death anxiety in different groups, observing varying anxiety levels among disease cohorts. Particularly noteworthy was the differing impact on anxiety levels across disease groups following exposure to thoughts of death. These findings suggest that death anxiety may be associated not only with emotional disorders but also with a broader spectrum of mental health factors, including paranoia, religious beliefs, depression, anxiety, stress disorders, and resilience in previous studies. We found that while an increase in income was associated with reduced death anxiety among individuals with COVID-19 pneumonia and healthy individuals, it did not affect those with MI. Additionally, we observed that an increase in education level was associated with higher death anxiety among MI patients but had no effect on individuals with COVID-19 pneumonia or healthy controls.

Furthermore, exposure to death-related news was associated with a decrease in Beck anxiety scores post-exposure, except in the MI group, where death anxiety was higher. This difference suggests that while the pandemic may create virtual anxiety and fear of death, such effects may not be present in diseases with a higher mortality rate like MI. Our findings also indicate an association between death anxiety and other psychopathologies, with exposure to death-related news during the pandemic exacerbating death anxiety. These observations underscore the influence of media on societal mental health during such events.

## Figures and Tables

**Table 1:** The demographic data of the population.

	COVID-19 Pneumonia *n* = 41	MI *n* = 41	Healthy *n* = 50	Total *n* = 132	*p*
Gender male/female	24/17	25/16	25/25	74/58	0.53 *
Age/mean ± SD	51.1 ± 16	63.5 ± 11	47.6 ± 14	53.7 ± 15	<0.001 **
Marital status (married %)	75.6	90.2	86	84.1	0.2 *
Education %: 8 years, 12 years, university	41.5	75.6	28	47	0.2 ****
	24.4	14.6	34	25	
	34.1	9.8	38	28	
Income %: low, medium, high	46.3	22	8	24.2	<0.001 ****
	34.1	73.2	42	49.2	
	19.5	4.9	50	26.5	
Working status %: working, unemployed, retired	43.9	17.1	52	38.6	0.001 ****
	34.1	24.4	26	28	
	22	58.5	22	33.3	
Following COVID-19 news %	58.5	56.1	22	43.9	<0.001 *
COVID-19-positive relatives %	87.8	73.2	94	85.6	0.01 *
COVID-19 anxiety/mean ± SD	2.27 ± 3.4	0.93 ± 1.7	0.92 ± 1.7	1.34 ± 2.4	0.01 ***
Beck anxiety/mean ± SD	15.8 ± 12.7	14.02 ± 9.1	9.3 ± 8.7	12.79 ± 10.5	0.008 ***
Thorson–Powell anxiety/mean ± SD	56.73 ± 10	57.02 ± 12.4	40.1 ± 9.4	50.52 ± 13.3	<0.001 **

MI: myocardial infarction. *p*-values were obtained from the *t*-test (*), ANOVA (**), Mann–Whitney U (***), and Kruskal–Wallis (****) tests, which were appropriate according to the distribution of data.

**Table 2:** Distribution and associations of anxiety levels.

		Thorson–Powell	Beck Anxiety	COVID-19 Anxiety
Gender	Male	52 (40–63.25)	8.5 (0–42)	0 (0–8)
	Female	50 (39.75–57.50) *p* = 0.244	13.5 (0–50) *p* = 0.004	0 (0–14) *p* = 0.966
Marital status	Married	51 (40–60)	10 (0–42)	0 (0–11)
	Single	52 (43–58.50) *p* = 0.796	12 (1–50) *p* = 0.211	0 (0–14) *p* = 0.230
Education	8 years	54 (45.25–62)	14.5 (0–46)	0 (0–11)
	12 years	46 (39.50–55)	9 (0–37)	1 (0–8)
	University	49 (37.50–60) *p* = 0.121 *	7 (0–50) *p* = 0.003 *	0 (0–14) *p* = 0.145 *
Income	Low	56 (51.25–62.75)	15 (0–46)	0 (0–11)
	Medium	51 (39.50–62)	11 (0–50)	0 (0–14)
	High	45 (37–51) *p* = 0.001 **	6 (0–24) *p* = 0.002 *	0 (0–8) *p* = 0.381 *
Working status	Working	48 (35–57)	8 (0–50)	0 (0–14)
	Unemployed	54 (44.50–62)	13 (0–46)	0 (0–11)
	Retired	52 (42.25–60) *p* = 0.124 *	14 (0–42) *p* = 0.008 *	0 (0–8) *p* = 0.298 *
COVID-19 news following	Yes	55 (44.75–66)	10 (0–50)	0 (0–14)
	No	48.50 (39–56) *p* = 0.003	11 (0–46) *p* = 0.965	0 (0–11) *p* = 0.108
COVID-19-positive relatives	Yes	51 (40–60)	11 (0–50)	0 (0–14)
	No	54 (48–67) *p* = 0.193	0 (0–37) *p* = 0.610	0 (0–8) *p* = 0.393
Population	COVID-19 pneumonia	56 (29–75)	12 (0–50)	0 (0–14)
	MI	60 (35–78)	13 (2–37)	0 (0–7)
	Healthy	42 (12–56) *p* < 0.001 *	8 (0–29) *p* = 0.008 *	0 (0–7) *p* = 0.078 *

MI: myocardial infarction. Mann–Whitney U was performed and median values are given in parenthesis. * Kruskal–Wallis test. ** Post hoc Tukey analyses showed that the difference was between medium and high income.

**Table 3:** The multivariate linear regression analysis between risk factors and anxiety levels.

		Thorson–Powell	Beck Anxiety	COVID-19 Anxiety
Gender	Female	-	6.96 (1.94) *p* < 0.001	-
Education	12 years	0.17 (2.68) *p* = 0.94	−3.27 (2.26) *p* = 0.15	0.38 (0.58) *p* = 0.5
	University	2.55 (3.01) *p* = 0.39	−5.77 (2.61) *p* = 0.029	0.27 (0.63) *p* = 0.67
Income	Medium	−2.91 (2.78) *p* = 0.29	2.48 (2.38) *p* = 0.29	0.67 (0.57) *p* = 0.24
	High	−2.34 (3.63) *p* = 0.52	−0.18 (3.18) *p* = 0.95	0.48 (0.75) *p* = 0.52
Working status	Unemployed	2.24 (2.79) *p* = 0.42	−1.94 (2.57) *p* = 0.45	-
	Retired	1.25 (2.72) *p* = 0.64	2.03 (2.31) *p* = 0.38	-
Following COVID-19 news	Yes	1.25 (2.11) *p* = 0.55	-	0.30 (0.46) *p* = 0.51
COVID-19-positive relatives	Yes	−0.28 (2.97) *p* = 0.92	-	-
Groups	MI	16.62 (2.90) *p* < 0.001	1.59 (2.35) *p* = 0.5	0.06 (0.61) *p* = 0.91
	COVID-19 pneumonia	15.14 (2.66) *p* < 0.001	6.86 (2.25) *p* = 0.002	1.48 (0.57) *p* = 0.011

Column variables shows beta estimate, standard error within parenthesis and *p*-values.

## Data Availability

The dataset used in this study is available on request. The data are not publicly available as they contain information that could compromise the privacy of research participants.
